# Enhanced Burst-Suppression and Disruption of Local Field Potential Synchrony in a Mouse Model of Focal Cortical Dysplasia Exhibiting Spike-Wave Seizures

**DOI:** 10.3389/fncir.2016.00093

**Published:** 2016-11-10

**Authors:** Anthony J. Williams, Chen Zhou, Qian-Quan Sun

**Affiliations:** Department of Zoology and Physiology, University of WyomingLaramie, WY, USA

**Keywords:** freeze lesion, microelectrode array, local field potential, cortical dysplasia, burst suppression, microgyrus, seizure

## Abstract

Focal cortical dysplasias (FCDs) are a common cause of brain seizures and are often associated with intractable epilepsy. Here we evaluated aberrant brain neurophysiology in an *in vivo* mouse model of FCD induced by neonatal freeze lesions (FLs) to the right cortical hemisphere (near S1). Linear multi-electrode arrays were used to record extracellular potentials from cortical and subcortical brain regions near the FL in anesthetized mice (5–13 months old) followed by 24 h cortical electroencephalogram (EEG) recordings. Results indicated that FL animals exhibit a high prevalence of spontaneous spike-wave discharges (SWDs), predominately during sleep (EEG), and an increase in the incidence of hyper-excitable burst/suppression activity under general anesthesia (extracellular recordings, 0.5%–3.0% isoflurane). Brief periods of burst activity in the local field potential (LFP) typically presented as an arrhythmic pattern of increased theta-alpha spectral peaks (4–12 Hz) on a background of low-amplitude delta activity (1–4 Hz), were associated with an increase in spontaneous spiking of cortical neurons, and were highly synchronized in control animals across recording sites in both cortical and subcortical layers (average cross-correlation values ranging from +0.73 to +1.0) with minimal phase shift between electrodes. However, in FL animals, cortical vs. subcortical burst activity was strongly out of phase with significantly lower cross-correlation values compared to controls (average values of −0.1 to +0.5, *P* < 0.05 between groups). In particular, a marked reduction in the level of synchronous burst activity was observed, the closer the recording electrodes were to the malformation (Pearson’s Correlation = 0.525, *P* < 0.05). In a subset of FL animals (3/9), burst activity also included a spike or spike-wave pattern similar to the SWDs observed in unanesthetized animals. In summary, neonatal FLs increased the hyperexcitable pattern of burst activity induced by anesthesia and disrupted field potential synchrony between cortical and subcortical brain regions near the site of the cortical malformation. Monitoring the altered electrophysiology of burst activity under general anesthesia with multi-dimensional micro-electrode arrays may serve to define distinct neurophysiological biomarkers of epileptogenesis in human brain and improve techniques for surgical resection of epileptogenic malformed brain tissue.

## Introduction

Malformations of cortical development lead to a diverse array of pathological brain disorders (Verrotti et al., 2010) with a high prevalence of refractory epilepsy and cognitive impairment (Guerrini and Dobyns, 2014). Focal cortical dysplasia (FCD) is a common form of cortical malformation that consists of discrete localized regions of disorganized cell structure and altered cortical lamination commonly associated with microgyric lesions (Crino, 2015). FCD can be induced by a lesion to the cortical surface early in development or from underlying genetic factors that alter cell migration patterns and disrupt the normal laminar formation of the cortex (Luhmann, 2016). Given the focal nature of FCDs, these patients are often candidates for surgical intervention aimed at removing the affected brain regions. In cases where seizures are drug resistant, surgical resection of the affected area may be the only recourse for severely affected patients (Sisodiya, 2000). Defining the epileptic zone is critical for surgical intervention and is an ongoing research area of interest (Sisodiya, 2000, 2004; Wendling et al., 2010). Complete resection of the epileptogenic zone is the strongest indicator of seizure-free outcome (Moosa and Gupta, 2014). Currently, neuroimaging techniques have been relied on as the best method for defining the extent of the lesion, with limited efficacy of neurophysiology recording to directly confirm hyperexcitable regions (Kabat and Król, 2012; Moosa and Gupta, 2014). Advances in the design and availability of high-density microelectrode arrays coupled with research aimed at discovering distinct electrophysiological biomarkers to define the epileptogenic zone may help to improve outcome in FCD patients, particularly in cases where the lesion is poorly defined by MRI or PET scans.

One of the primary experimental animal models for studying FCD is the neonatal freeze lesion (FL) model (Dvorák and Feit, 1977; Dvorák et al., 1978; Rosen et al., 1992; Luhmann and Raabe, 1996) that exhibits similar pathology, epileptic disturbance, and cognitive dysfunction to FCD in humans (Kamada et al., 2013; Luhmann, 2016; Sun et al., 2016). Acute FLs to the neonatal rodent cortex at P0 result in distinct structural abnormalities due to disrupted cellular migration patterns and repair of the injured cortex (Rosen et al., 1992) leading to a distortion of apical pyramidal neuron morphology, disorganization of cells in the deep cortical layers below the lesion, and inflection of apical dendrites towards the microsulcus (Hagemann et al., 2003). The resulting dysplastic cortex is characterized by a stereotypical inward “dimpling” of the cortical surface consisting of a 3–4 layered dysplastic cortex surrounding a deep involution of the induced microgyrus in the adult cortex (Rosen et al., 1992; Luhmann and Raabe, 1996). Depending on the severity and number of FLs, the induced pathology can vary from mild ectopic lesions to the formation of a deep microgyric cleft (schizoencephaly) and associated porencephalic cysts, similar to the spectrum of pathology observed in human FCD (Rosen and Galaburda, 2000). Though the microgyrus and associated dysplastic region defines the core histological abnormality of the freeze-lesioned brain, alterations in afferent/efferent projections, cell physiology, and receptor expression can extend well beyond the dysplastic center into the paramicrogyral zone of the ipsilateral cortex (Zilles et al., 1998; Jacobs et al., 1999b; Rosen and Galaburda, 2000) as well as altered physiology in remote brain regions as distant as the contralateral somatosensory cortex (Schmidt et al., 2006).

The epileptogenicity of human and animal models of FCD has been attributed to an imbalance in the level of excitation to inhibition in the injured cortex (Redecker et al., 1998; Avoli et al., 1999; Zhu and Roper, 2000), a phenomena underlying general disturbances to the brain including epilepsy (Eichler and Meier, 2008). *In vitro* slice studies have indicated that experimental FCDs induce a hyper-excitable region of tissue thought to arise from a hyper-innervation of thalamocortical projections that have lost their original targets within the lesion and shift their inputs to neighboring cells (Jacobs and Prince, 2005). This hyper-excitable zone of brain tissue can typically extend up to several mm from the core microgyric lesion (Jacobs et al., 1996, 1999b; Luhmann and Raabe, 1996; Roper et al., 1997; Redecker et al., 1998). In contrast to the hyperexcitability observed in brain slices induced by experimental FCD, behavioral or electrographic seizures are not always present *in vivo* (Kellinghaus et al., 2007) at least without a provoking event (Luhmann, 2016). The lack of reports of electroencephalogram (EEG) seizures in FCD models may be related to a variety of factors including the location or severity of the induced FCD, the possible non-convulsant nature of the seizure, the prevalence of the induced seizurogenic activity, or the timing of seizure onset. Despite these concerns, two rodent FL models have reported prevalent spontaneous electrographic seizures in adult animals. The first involves inducing early bilateral FLs to the scalp of unborn rats at day E18 through the uterine wall, resulting in robust hippocampal spike/polyspike discharges in up to 69% of animals tested (Takase et al., 2008; Kamada et al., 2013). More recently, a mouse model utilizing single unilateral FL to the S1 cortex (the model used in the current study) has been associated with a high prevalence of spontaneous non-convulsant spike-wave discharges (SWDs) in mature adults (>5 months old) occurring primarily during sleep (Sun et al., 2016). The presence of spontaneous electrographic seizures following neonatal FLs also tends to worsen outcome as cognitive deficits are more severe in FL animals with seizures than those without Sun et al. (2016).

In the current study, we employ linear depth electrode microarrays to probe the aberrant neurophysiological structure of cortical and subcortical changes in extracellular potentials from the FL and normal brain. Specifically, we explore changes in the extracellular field potential across anesthesia level as compared to continuous cortical EEG recordings in both anesthetized and unanesthetized states in the same animal. Utilizing silicon microelectrode probes (Smartprobe electrode arrays, NeuroNexus), we were able to capture simultaneous extracellular epochs of spatiotemporal brain activity across cortical lamina and underlying subcortical structures as related to alterations in local field potentials (LFP), multi-unit activity (MUA), power spectra and signal synchrony. The main finding from these studies indicate an increase in the prevalence and severity of anesthesia-induced burst/suppression activity in the FL brain that share similar spike/wave components with the SWDs observed in unanesthetized animals. Technological advances in the miniaturization, stability, resolution and commercial availability of low noise multi-electrode recording allows for better access to mapping of multi-dimensional electrographic activity in animal models of disease and will open new paths of research aimed at describing the underlying neural circuits that drive aberrant brain activity (Berényi et al., 2014) with hopes to map entire neural networks in the near future (Alivisatos et al., 2012). Identification of viable neurophysiological biomarkers for establishing the region of hyperexcitable tissue during pre-surgical mapping of affected brain regions in FCD patients may also help to improve outcome aimed at surgical removal of the epileptogenic zone.

## Materials and Methods

### FL Model

Unilateral single or multiple FLs were made in P0–1 mice pups (male and female on a CD-1 background) to induce neocortical microgyria to the right cortical S1 area, using a modification of the method by Dvorák and Feit (1977) as previously described in detail (Sun et al., 2016). Control animals included a sham surgery (exposed to all surgical procedures minus the FL). Briefly, mice pups were immersed in wet ice for approximately 2 min until movement and response to tail pinch was absent. The skull was then exposed through a midline scalp incision and a freezing probe with a 1 mm diameter circular tip (cooled to −196°C in liquid nitrogen) was placed on the skull over the somatosensory cortex, 2 mm lateral to midline and 0.15 mm caudal to the bregma for 3 s. The scalp incision was closed with surgical glue, the pup warmed, and returned to the dam. This procedure was routinely completed within 5 min and resulted in the development of a consistent microgyrus in the adult brain (Sun et al., 2016). The mice were then weaned and housed together based on sex in a vivarium maintained at 22–23°C on a 12:12 h light-dark cycle. Food and water were available *ad libitum*. Brain physiology was evaluated in mature adults starting at 5–13 months of age using either acute extracellular recordings with linear micro-electrode arrays in anesthetized mice and/or cortical EEG electrode implantation and 24 h recording of anesthetized or non-anesthetized EEG in freely behaving animals. Following electrophysiological recordings animals were euthanized and brain tissue collected for histological analysis.

### Microelectrode Arrays

Extracellular signals were recorded using linear microelectrode SmartProbe^TM^ arrays (NeuroNexus, Ann Arbor, MI, USA) from the S1 region of control and FL mice. The electrode array consisted of a single shank with 16 individual electrodes separated by 100 μm with on-board electronics for digital conversion of the signals and linked to a SmartBox^TM^ control and data streaming system through a SmartLink headstage (NeuroNexus). The array was positioned perpendicular to the cortical surface to allow recording from both cortical and subcortical brain structures. Each signal was digitally filtered (1–10,000 Hz band-pass and 60 Hz notch filters), and recorded at a sample rate of 20 kHz using Smartbox 2.0 software (NeuroNexus). Additional off-line digital filtering was used to define LFPs (1–100 Hz) and MUA (300–3000 Hz).

### Extracellular Recordings

Prior to extracellular recording, mice were isolated and anesthetized with isoflurane (2%, delivered in oxygen) and placed in a stereotaxic frame (with mouse and gas anesthesia adaptors, Stoelting, Wood Dale, IL, USA). Core body temperature was maintained normothermic during all procedures with a circulating water bath (Haake, Thermo Electron, Newington, NH, USA) infused custom heating pad and continuous rectal temperature monitor (BK precision, Yorba Linda, CA, USA). A scalp incision was made to expose the skull over the right S1 area. A custom built stainless steel head bar was glued to the skull with dental cement to stabilize the head during recording. Once the dental cement cured, the animal was transferred to a custom-designed stage and the head bar securely clamped to allow ease of access with the recording electrodes. The recording stage was located within a custom-designed Faraday cage on a compressed-air stabilized table (Vibraplane, Kinetic Systems, Boston, MA, USA). All electrical recording devices were located outside of the Faraday cage to minimize electrical noise and all metal components within the cage connected to a common ground. A nose cone was used to deliver a continuous flow of isoflurane anesthesia (0.5–3.0%) during the recording session. Single or multiple burr holes (1 mm diameter) were made through the skull with a dental drill for insertion of linear micro-electrode arrays. Periodic administration of warm physiological saline was administered to the craniotomy site during recording. A reference electrode (silver wire) was placed in the skin flap of the scalp incision. The micro-electrode array was then positioned over the craniotomy at an angle of 40° from vertical for perpendicular insertion into the S1 cortex. A hydraulic micromanipulator (Narishige, Amityville, NY, USA) was used to slowly advance the array into the brain and allowed to stabilize for several minutes once in place (~1650 μm depth). Electrode placement was visually guided under a dissecting microscope to verify that all electrodes penetrated the surface of the brain. The dura was found to offer only minimal resistance to electrode insertion so was left intact. Extracellular recordings began once stable baseline activity was observed.

### Intracranial EEG

A single intracranial bipolar cortical electrode was chronically implanted over the S1 cortex to allow continuous EEG recordings in freely behaving animals as previously described (Sun et al., 2016). In some animals, cortical electrodes were implanted immediately following extracellular recordings. For EEG electrode implantation, mice were anesthetized under isoflurane anesthesia (2%, delivered in oxygen) and secured in a stereotaxic frame (with mouse and gas anesthesia adaptors, Stoelting, Wood Dale, IL, USA). Polyimide-insulated stainless steel wires (125 μm diameter, California Fine Wire Co. Grover Beach, CA, USA) and connecting pins (Digikey, Thief River Falls, MN, USA) were implanted over the ipsilateral S1 region near the site of extracellular recordings and FL. A reference electrode was placed into the ipsilateral olfactory bulb area. A screw free, glue-based electrode assembly system that allows for long-term recordings was used for all EEG recording sessions (Wu et al., 2008). Mice were returned to the vivarium after EEG electrode implantation and allowed to recover. EEG recordings were performed over a 24 h cycle with automated infrared (IR)-activity tracking to detect animal movement. During recording, animals were able to move freely in a recording chamber supplied with water gel. A bundle of soft and light-weight fine wires connecting the screw-free EEG electrodes to the recording amplifiers was anchored on the center of a circular recording arena that allowed for free movement of the animal. EEG signals were amplified (Model 1700 differential AC amplifier, A-M system, Carlsborg, WA, USA), filtered (1–100 Hz band-pass and 60 Hz notch filters), digitized (Power 1401 A-D converter, Cambridge Electronic Design Limited, Cambridge, England), and continuously recorded for 24 h (Spike2 software, Cambridge Electronic Design Limited). EEG recordings were performed either in awake, freely moving animals over a 24 h period or in anesthetized conditions. For anesthetized EEG, the recording chamber was sealed and isoflurane (0.5%–3.0% delivered in oxygen) was perfused into the chamber. Initially, anesthesia was induced at 3.0% isoflurane. Once the animal stabilized the anesthesia level was gradually lowered (2.0%, 1.0%, and 0.5%) with a 15 min recording at each level of anesthesia. The anesthetic was then stopped and continuous recordings continued for 8–10 h as the animal recovered.

### Histology

Following electrophysiology recordings, animals were anesthetized and transcardially perfused with 0.9% saline followed by 4% paraformaldehyde. Brains were extracted, immersed in 4% paraformaldehyde for 24 h, and then transferred to 0.1 M phosphate buffer containing sequentially increasing levels of sucrose (10/20/30%, pH 7.4, 4°C) across three consecutive days. Brain issue was then removed from the sucrose solution and cut into serial sections through the area of interest. The back of the micro-electrode was swabbed with DiI (2 mg/ml in ethanol, Invitrogen Molecular Probes, Eugene, OR, USA) for visualization of the electrode track in histopathological sections. Several techniques were used to visualize the probe track in relation to the FL and S1 barrel field. Thick coronal sections (300 μm) were cut on a vibratome for direct visualization of the probe track (DiI) in relation to the FL. A cryostat (Microm, Thermo Fisher Scientific, Kalamazoo, MI, USA) was used to cut thinner coronal sections (50–80 μm) for staining with cytochrome oxidase to visualize the barrel cortex in relation to the FL. In a subset of samples, the right and left cortices were carefully removed and flattened for topographic visualization of the entire barrel field (Jiao et al., 2006). Coronal or flattened brain sections were incubated at room temperature in a solution of cytochrome oxidase (0.5 mg/ml, Sigma), sucrose (0.4 mg/ml, Sigma), and DAB (0.625 mg/ml, Sigma) in 0.1 M phosphate buffer (pH 7.4) for 60–90 min until cortical barrels were visible under a dissecting microscope. All sections were then mounted on glass slides with a DAPI mounting medium (Vectashield, Vector Laboratories, Burlingame, CA, USA) and cover-slipped. Brain images were evaluated under a light/fluorescent microscope (Zeiss Axioskop 2, Ontario, CA, USA) and digitally imaged using Axiovision software (Version 4.6, Zeiss). Histological analysis of serial brain sections or flattened cortical images was used to construct cortical maps to mark the location of the recording sites in relation to the induced microgyric lesions.

### Data and Statistical Analysis

All extracellular signal files recorded using linear micro-arrays were exported to NeuroExplorer (Nex Technologies, Madison, AL, USA) for data analysis and visual inspection. In each case, no significant effects were contributed to the sex of the animal so data for males and females were combined. Quantitative analysis of discrete extracellular recording samples (2 s duration) were analyzed for changes in the spectral profile including power spectral density (PSD) and LFP synchrony (i.e., cross-correlation between signals). PSD values were computed across a frequency range of 1–20 Hz with a single taper Hann FFT using a bin size of 0.0012 Hz and 50% overlap between bins to mitigate data loss at the spectral edge of each bin. Correlation analysis was used to compute the cross-correlation between simultaneously recorded signals from a single linear microarray recording using standard correlograms across a −0.3 to +0.3 s offset (bin size of 0.05 ms based on the A/D sampling rate). Signals with similar waveform patterns exhibit high correlation values (ranging from 0 to 1) with negative values representing a reversal in phase of the recorded signals. For comparison, the cross-correlation values of all channels were compared to the cortical recording from channel 5 (approximate level of the granular layer, i.e., see Figure 3).

Following analysis, raw data was exported to Microsoft Excel for tabulation of statistical averages and standard error values or Sigmaplot (ver. 11.0, SYSTAT software Inc., San Jose, CA, USA) for graphical display and statistical analysis between groups. Group averages are presented mean ± SEM. Student *t*-tests were used to evaluate significant differences between two groups. A multifactorial analysis of variance (ANOVA) was used to evaluate main effects and interactions when multiple independent variables were present including electrode depth, anesthetic state, treatment group (e.g., control vs. FL), or animal sex, followed by a Holm-Sidak *post hoc* analysis to evaluate significant differences between individual groups. Three main variables were evaluated: incidence of burst suppression, PSD values, and signal synchrony. A Kaplan-Meier Log-Rank analysis was used to evaluate the incidence of burst/suppression in control and FL animals across anesthesia level. Pearson’s correlation was used to determine the relationship between cortical vs. subcortical signal cross-correlation values and the distance to the microgyrus. A *P* value of <0.05 was considered significant.

Intracranial EEG signals were visually inspected offline for the presence of epileptiform activity. The following seizure parameters were examined across the 24 h recording period: total number of seizure spike-train events, total seizure duration, average number of spikes per seizure event. The timing of epileptiform activity was plotted against specific sleep stages (REM, NREM, Wake, and Active Awake) using methods previously described (Sun et al., 2016). Briefly, sleep-stage detection was based on a Fast Fourier Transform of the EEG signal from 1 Hz to 20 Hz, yielding power in δ, θ, α, σ and β bands plus integrated floor sensor activity across 2-s epochs. REM sleep was identified by an increased θ power and concomitant low δ and σ power in association with a lack of movement. Changes in δ power were used to distinguish NREM sleep from quiet waking epochs (without movement) or active awake epochs (with movement).

## Results

Extracellular potentials were recorded simultaneously from 16 electrodes (using linear micro-arrays) in anesthetized animals to compare spontaneous extracellular waveforms across electrode depth and anesthetic state in both control and FL animals. A total of 17 adult mice were evaluated (eight control and nine FL) ranging in age from 5 to 13 months old. The average age of control (mean = 6.44 ± 0.65 months) and FL (mean = 8.16 ± 0.97 months) animals were not significantly different (*P* > 0.05, *t*-test). Control animals included both normal mice (*n* = 4) and those exposed to a sham surgery (*n* = 4). Quantitative endpoints included the incidence of burst activity, PSD values, and signal cross-correlation. Individual extracellular recordings typically lasted 1–2 min in duration with multiple recordings obtained for each animal across anesthetic states and at different locations within the S1 cortex. Upon completion of electrophysiological recordings, brain tissue was collected for histological comparison of electrode recording site to microgyrus location. Extracellular recordings were also compared to spontaneous cortical EEG waveforms in unanesthetized animals.

### FL Injury to S1 Cortex

**Figure 1:**
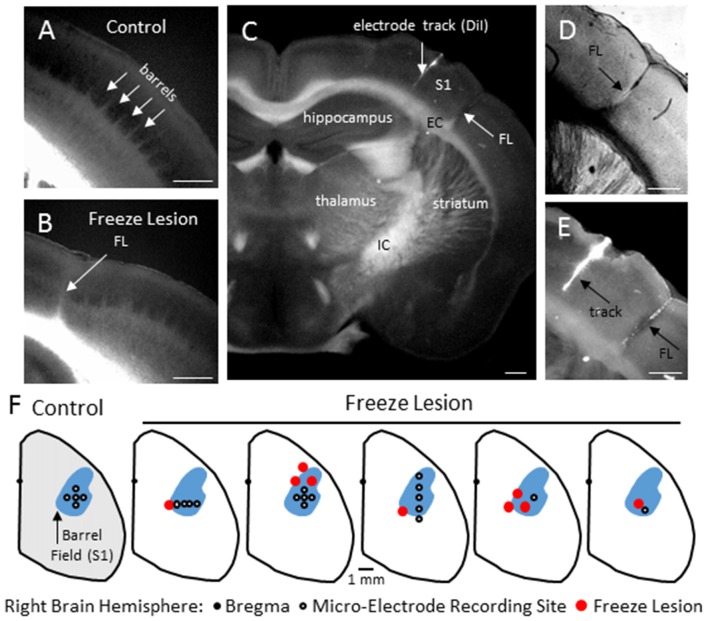
**Freeze Lesion (FL) histopathology. (A)** Normal barrel formation in the S1 cortex of a control mouse (cytochrome oxidase stain). **(B)** Disrupted barrel formation and presence of cortical microgyrus in a FL mouse brain. **(C)** Coronal hemisphere of a FL brain referencing the S1 cortex and underlying subcortical structures including the internal (IC) and external (EC) capsules. DiI was applied to the shaft of the linear microarray to mark the electrode track position in comparison to the FL (arrows). **(D,E)** Higher magnification images of the S1 cortex shown in panel **(C)** showing an example of FL induced microgyrus **(D)** and labeled recording track **(E)**. **(F)** Position of the microelectrode in comparison to the S1 region and FL microgyrus was mapped in four controls and five FL animals. In control animals, recordings were made at one or more of the electrode positions as shown. In FL animals, each cortical map is shown indicating the electrode position in relation to the microgyrus. The barrel field (shown in blue) was identified using a cytochrome oxidase stain. White scale bars in panels **(A–E)** indicate 0.5 mm.

Neonatal FLs disrupted normal S1 whisker barrel formation, as indicated from cytochrome oxidase staining (Figure 1B) as compared to the normal barrel structure in control brains (Figure 1A). Flattened cortical brain sections were also evaluated (i.e., see Figures 8B, 9B) that indicated a disruption of normal barrel formation in the region immediately surrounding the FL, similar to previous reports (Jacobs et al., 1999c). In the remaining tissue samples, coronal sections of FL brains indicated a deep involution of the microgyrus extending across all cortical layers (Figure 1C, expanded in Figure 1D). DiI staining was used to assess the distance of the recording electrode to the nearest microgyrus (Figure 1C, expanded in Figure 1E). The position of the micro-electrode was mapped in reference to the S1 barrel field and FL microgyrus in four controls and five FL animals (Figure 1F). All recording sites were located within the barrel field (Figure 1F). The location of the FL microgyria varied across animals but was confined to the SI region in all animals studied.

### Effects of Isoflurane Anesthesia on Extracellular Potentials

**Figure 2:**
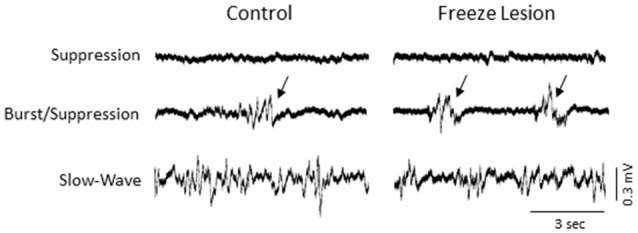
**Effect of isoflurane-mediated anesthesia on extracellular recordings.** Three anesthetic states were defined by the presence of suppression, burst/suppression, or slow-wave activity in extracellular recordings from the S1 cortical region of both control and FL animals. Signals are presented at the same scale to compare changes in signal amplitude. Black arrows indicate burst activity.

The level of isoflurane anesthesia induced stereotypical waveform patterns in extracellular recordings from the S1 brain region of both control and FL animals that changed as the anesthetic dose was reduced (Figure 2), representing the transition from a deep (stage IV) to a lightly anesthetized state (stage III-3), similar to that observed from LFP recordings in ferrets (Sellers et al., 2013) and from electrocorticogram recordings in rats under halothane or urethane anesthesia (Friedberg et al., 1999). Three anesthetic states were defined based on the dominant waveform observed in the extracellular recording: suppression, burst/suppression, and slow-wave activity (Figure 2, see “Discussion” Section). The deepest level of anesthesia induced a general suppression of the extracellular signal (Figure 2, top traces) with loss of toe pinch withdrawal and vibrissal movement, observed in all animals at a 3% or higher level of isoflurane. As the anesthetic dose was lowered (0.5–2.0%) the suppressed waveform was interrupted by brief episodes of arrhythmic burst activity (Figure 2, middle traces). Respiration rate increased as the anesthetic level was lowered with occasional toe pinch withdrawal, particularly at 0.5–1.0% isoflurane. Distinct volleys of burst activity occurred in a pseudo-rhythmic pattern with each burst typically lasting only a few seconds in duration (Figure 2, middle traces; see below). At lower levels of anesthesia, a continuous pattern of high amplitude slow-wave activity was the predominant waveform, observed in all animals at 0.5% isoflurane or lower. This lightly anesthetized waveform was termed “slow-wave” based on the predominant low-frequency (1–4 Hz) structure of the waveform (see power analysis below) similar to that reported in the cortical EEG of lightly anesthetized animals or during slow-wave sleep (Friedberg et al., 1999).

### Burst/Suppression

**Figure 3:**
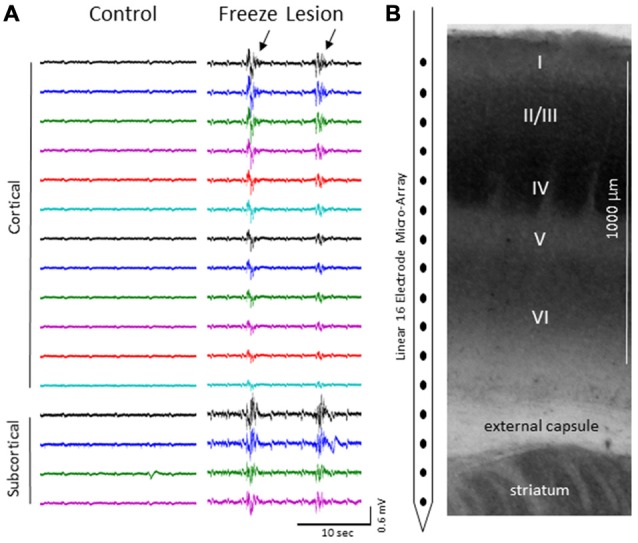
**(A)** Representative micro-array recordings from the S1 cortex of a control and FL animal under 2.0% isoflurane anesthesia. Black arrows indicate presence if burst activity. **(B)** Diagram of the 16 electrode array in comparison to the cortical lamina of the S1 region (layers I-VI) and underlying subcortical brain structures.

We focus the main part of this study on burst activity as burst/suppression is generally considered a hyper-excitable brain state induced by gas anesthetics (Amzica, 2009) and the FL brain has been shown to be prone to hyper-excitable evoked activity near the site of the FL (Jacobs et al., 1996; Luhmann et al., 1998; see “Discussion” Section). Burst activity was typically observed across all cortical lamina and subcortical structures of control and FL animals (Figure 3) though differences in signal amplitude and spectral profile of individual bursts could vary, particularly between cortical and subcortical regions of FL animals (see PSD and LFP synchrony sections below). Note the abrupt change in signal strength of burst activity near the transition from the cortex to subcortex (e.g., channel 13) that was subsequently used as a physiological marker to verify electrode depth (Figure 3). Bursts typically lasted 1–3 s in duration (control = 2.32 + 0.27 s, FL = 1.92 + 0.17 s, *P* > 0.05 between groups, *t*-test) at a frequency of 1–12 bursts over a 60 s epoch (0.017–0.2 Hz) and occurred near simultaneously across the entire vertical depth of the 16 electrode microarray with no indication of a focal/laminar region of origin in any of the animals studied.

**Figure 4:**
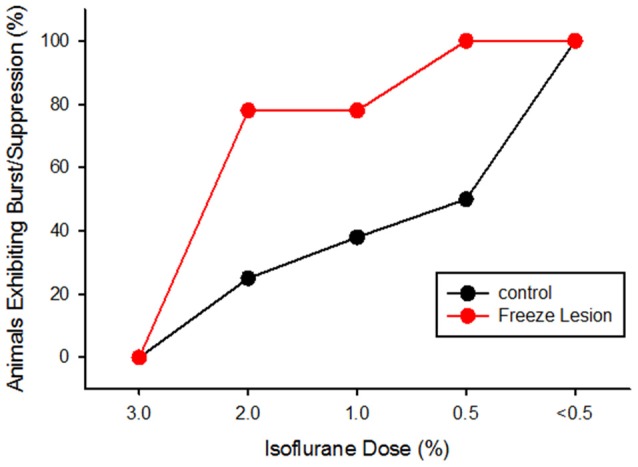
**Cumulative activity graph of burst/suppression activity in control and FL animals (*P* < 0.05 between groups, Kaplan-Meier Log-Rank test).** Data are presented as the percent of animals that exhibited burst/suppression as the level of isoflurane anesthesia was lowered from 3.0% to 0.5%.

The incidence of burst/suppression between control and FL animals across anesthesia level is presented in Figure 4. Data was collected from eight controls and nine FL animals. The incidence of burst/suppression was recorded as the level of isoflurane anesthesia was reduced from 3.0% to 0.5%. In comparison, the incidence of burst/suppression was significantly higher in FL animals as compared to controls (Figure 4, *P* < 0.05, Kaplan-Meier Log-Rank test). Median onset of burst/suppression in control animals occurred at 0.5% isoflurane. Median onset of burst/suppression in FL animals occurred at 2.0% isoflurane. No significant differences were observed between male and female mice (*P* > 0.05) or between normal and sham-treated controls (*P* > 0.05, Kaplan-Meier Log-Rank test). The presence of burst activity was based on evaluation of multiple recordings in each animal. Average extracellular recording times between control (4.3 ± 1.1 min) and FL (5.6 ± 1.4 min) animals were not significantly different (*P* > 0.05, *t*-test). In animals exhibiting burst/suppression, there was a significant increase in the number of bursts/min across anesthetic dose (*P* < 0.05) but not between control and FL animals (*P* > 0.05, ANOVA). The average number of bursts/min increased from 3.32 ± 0.99 (2.0% isoflurane) to 7.01 ± 1.42 (1.0% isoflurane) and 6.80 ± 1.26 (0.5% isoflurane) across anesthesia levels. Although there were no gender specific differences in the data reported in the current study, long term EEG studies in awake mice revealed that SWDs are more prevalent in female mice (Sun et al., 2016). Gender differences in hyperexcitable extracellular waveforms may not have been detected due to the lower group size in the current study (*n* = 17) and compared to the previous EEG study (*n* = 45; Sun et al., 2016). Further studies will be needed to address the possible roles of sex-specific incidence of seizures following FCD.

**Figure 5:**
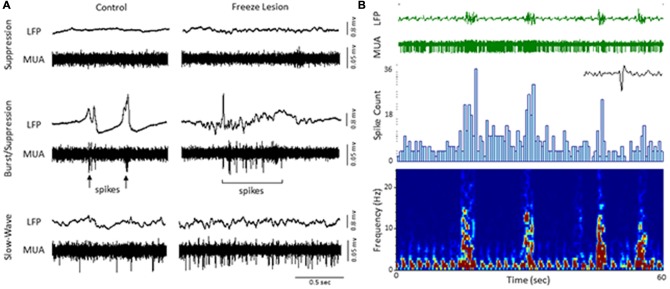
**Local field potential (LFP, 1–100 Hz) and multi-unit activity (MUA, 300–3000 Hz). (A)** Representative extracellular waveforms (LFP, top) from a control and FL mouse indicating the change in spontaneous cortical spiking (MUA, bottom) dependent on anesthetic state. Each large deflection in the MUA signal (black arrows) represented a spontaneous spike from a cortical neuron. **(B)** Burst/suppression recording from a FL animal. An expanded spike waveform is shown to the lower right of the MUA signal. LFP bursts were typically associated with an increase in both spike count (spike histogram, middle panel) and spectral power (spectrogram, lower panel).

An increase in spontaneous single-unit spiking was also observed during the presence of burst activity (Figure 5). To assess the effects of burst activity on single-unit responses, raw extracellular signals were digitally filtered to extract LFP (1–100 Hz) from MUA (300–3000 Hz). Spontaneous cortical or subcortical spiking was occasionally observed in one or more channels of the linear microarray, in either control or FL animals, as indicated by large amplitude deflections in the MUA signal. Two representative examples of LFP vs. MUA activity from a control and FL animal at different levels of anesthesia are shown in Figure 5A. At the deepest level of anesthesia, associated with signal suppression, neuronal spiking was rare (Figure 5, top row). As the anesthesia level was lowered, the presence of burst activity (predominately confined to large amplitude fluctuations in the LFP) was often associated with the appearance of neuronal spiking (Figure 5A, middle row). It was not uncommon to see spontaneous spiking during slow-wave activity as well (Figure 5A, bottom row). Figure 5B demonstrates the change in activity of a spontaneously firing neuron during alternating levels of burst/suppression activity of a FL animal. Spike count increased during periods of burst activity (Figure 5B, middle row) along with an increase in spectral power of the recorded signal (Figure 5B, bottom row).

### Power Spectral Density

**Figure 6:**
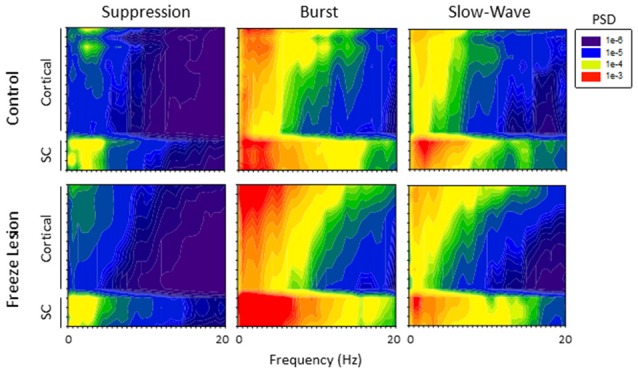
**Power spectral density (PSD).** Spectrogram plots of the change in PSD across anesthetic state of control and FL animals. The spectral plots shown here were constructed from the average PSD values of multiple traces across all animals in each group.

PSD analysis was used to evaluate changes in the frequency spectrum of extracellular signals across electrode, anesthesia state, and treatment group (control vs. FL). Spectrogram plots of the change in average PSD values across anesthetic state and treatment group are shown in Figure 6. Suppression was associated with the lowest levels of PSD in the recorded signals, predominately confined to the lower frequencies (1–4 Hz; Figure 6). It was not uncommon to observe a 10 to 100-fold increase in PSD values during brief periods of burst activity, including increases in the higher frequency, theta-to-alpha band regions (4–12 Hz; Figure 5). Individual spectral plots of burst activity generally consisted of 1–4 spectral peaks ranging in peak frequency between 1–8 Hz in both control and FL animals. The highest levels of burst PSD were observed in the superficial cortical layers and subcortical structures (Figure 6). As the anesthetic level was lowered, slow-wave activity tended to maintain a similar PSD profile as compared to burst activity though at lower power levels (Figure 6). Regardless of anesthetic state the majority of PSD values were confined to the 1–20 Hz spectral range. In comparison, both control and FL animals exhibited a similar change in the spectral profile of PSD across anesthesia levels, however, FL animals exhibited a greater increase in PSD during burst activity (see below).

**Figure 7:**
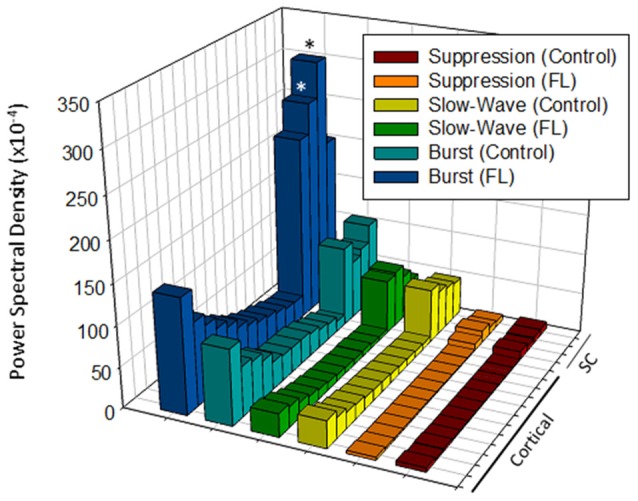
**Comparison of average PSD values across groups (total power, 1–20 Hz).** Significant differences were measured across the treatment group, anesthetic state and electrode (*P* < 0.05, analysis of variance (ANOVA), see “Results” Section). *P < 0.05 between control and FL (Holm-Sidak *post hoc* analysis).

A comparison of the quantitative difference in total power (1–20 Hz) across electrodes, anesthesia state, and treatment groups is presented in Figure 7. Data was collected from a total of eight controls and nine FL animals. Multifactorial ANOVA indicated significant main effects across all three independent variables (*P* < 0.05) though differences in PSD values across both electrodes and treatment group were dependent on anesthesia level (*P* < 0.05 interaction between groups). A Holm-Sidak *post hoc* analysis was used to measure significant differences across electrodes and treatment groups at each level of anesthesia: *Electrode*: within group analysis indicated significant differences in PSD values between cortical and subcortical electrodes. Specifically; subcortical electrodes 13 in control and 14 in FL (suppression), 13–15 in FL (burst), 13 in control and 13–15 in FL (slow-wave) groups had significantly higher PSD values than in one or more cortical electrodes (*P* < 0.05 Holm-Sidak *post hoc* analysis). Although there appeared to be a trend towards an increase in PSD values in the superficial cortical layers (i.e., electrodes 1–3), the individual groups values were not statistically different than the deep cortical layers (*P* > 0.05). *Treatment Group*: significant differences between individual treatment groups were only observed during burst activity. Specifically, PSD values from subcortical electrodes 14 and 15 of FL animals were 3.7 and 3.8-fold higher (respectively) than in control animals (Figure 7, *P* < 0.05, Holm-Sidak *post hoc* analysis). This data suggests that although the malformations are in the cortex, subcortical activities and PSDs carried valuable information regarding excitable brain states.

### Extracellular Signal Synchrony

**Figure 8:**
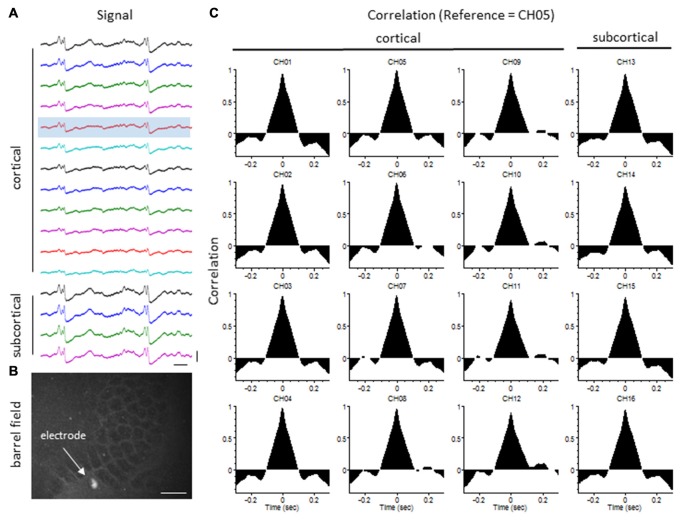
**LFP synchrony in control animals. (A)** Representative recording from a control animal indicating the similarity in burst waveform across cortical and subcortical layers despite the change in signal amplitude. Recording channels are presented in sequential order from cortical surface (CH01) to the deepest subcortical electrode (CH16). Channel 5 (shaded) was used as the reference channel for the cross-correlation analysis. Vertical scale bar = 0.4 mV, horizontal scale bar = 100 ms. **(B)** Location of the recording electrode within the cortical barrel field (flattened cortex, cytochrome oxidase staining). White scale bar = 0.5 mm. **(C)** Cross-correlation values. Note the high correlation values (near 1) across all electrodes occurring at time = 0 (i.e., no offset between signals).

**Figure 9:**
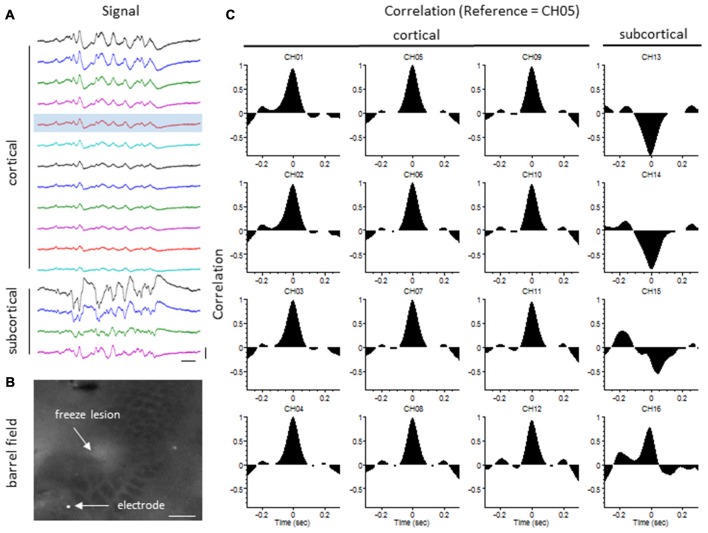
**LFP synchrony in FL animals. (A)** Representative recording from a FL animal indicating the change in burst waveform across electrodes, specifically between cortical and subcortical layers. Recording channels are presented in sequential order. Channel 5 (shaded) was used as the reference channel for the cross-correlation analysis. Vertical scale bar = 0.4 mV, horizontal scale bar = 100 ms. **(B)** Location of the recording electrode in reference to the FL microgyrus (flattened cortex, cytochrome oxidase staining). White scale bar = 0.5 mm. **(C)** Cross-correlation values. A strong correlation between electrodes was observed across cortical layers but were out of phase in subcortical layers as indicated by the negative shift in the cross-correlation values at time = 0 (CH13–15).

**Figure 10:**
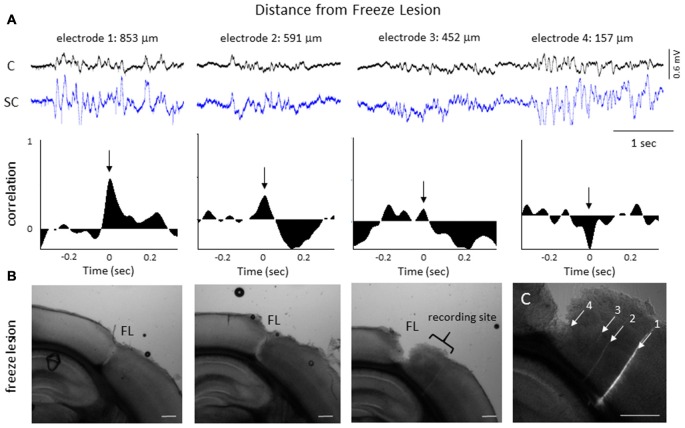
**LFP phase shift at different distances to the FL induced microgyrus. (A)** Representative FL animal, indicating the change in signal cross-correlation between cortical and subcortical signals as the electrode was moved closer to the FL microgyrus. Cortical (CH05) and subcortical (CH13) burst signals are presented from four recording sites near the FL microgyrus (upper row). Distance to the FL is shown above each signal. The associated cross-correlation values for each recording site is shown directly below the raw signals (middle row). Note the shift in the correlation values from positive to negative as the recording electrode approached the microgyrus (black arrows, time = 0). **(B)** Three successive serial sections from the brain of this animal indicating the site of the FL microgyrus in comparison to the recording site. White scale bars = 0.5 mm. **(C)** Expanded image of the recording site showing the position of the recording electrodes as determined from DiI staining. The numbered tracks correspond to the recorded signals shown in the panels above. White scale bar = 0.5 mm.

The degree of synchrony between simultaneously recorded extracellular signals was determined by cross-correlation analysis (see “Materials and Methods” Section). Here we focus on signal synchrony of extracellular bursts across cortical and subcortical layers. Visual examination of the recorded burst signals typically indicated highly synchronous waveform patterns across the entire linear depth of the microarray including cortical and subcortical regions except near the site of the FL. To quantitate the degree of synchrony between the recorded signals, channel 5 was chosen as the reference channel (i.e., the cross-correlation value for each electrode was compared to channel 5). A typical pattern of signal correlation across electrodes is shown in a representative control animal in Figure 8. In this animal, a high degree of signal synchrony was observed across all cortical and subcortical layers as indicated by the similar pattern of the cortical bursts recorded from each electrode (Figure 8A). Electrode position within the S1 region is shown in the flattened cortex image of Figure 8B. Individual cross-correlation plots from each channel (referenced to channel 5) indicated a strong correlation across all electrodes (as indicated by high correlation values near 1.0) with maximal values near time = 0 (indicating a minimal temporal offset between signals; Figure 8C). FL animals exhibited a different pattern of signal synchrony between electrodes as demonstrated by the extracellular recording of the FL animal in Figure 9. In this animal, there was an abrupt change in the recorded subcortical signal pattern as compared to cortical signals (Figure 9A). A comparison of the recording location to the FL-induced microgyrus is shown in Figure 9B. Cross-correlation analysis indicated a high degree of synchrony in cortical signals, similar to control animals, however, a strong negative correlation is seen in subcortical recordings (channels 13–15) indicating a reversal in phase of the recorded signals (Figure 9C). Further analysis in FL animals indicated that signal synchrony between cortical and subcortical signals weakened as the recording electrode approached the FL with eventual phase reversal near the site of the microgyrus (Figure 10).

**Figure 11:**
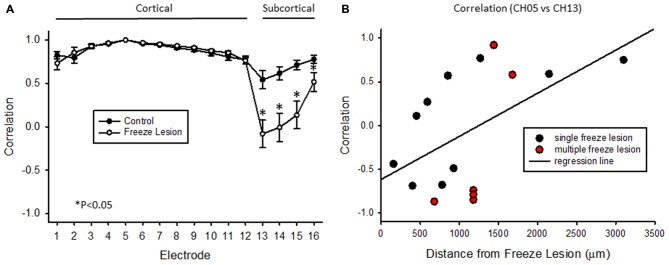
**Cross-correlation values between cortical and subcortical signals. (A)** Average cross-correlation values across all 16 micro-electrode recordings, in reference to channel 5. Significant differences were measured between control and FL animals (*P* < 0.05, ANOVA) dependent on electrode position (*P* < 0.05, interaction between variables). Data are presented as the mean ± SEM. *P < 0.05 between control and FL (Holm-Sidak *post hoc* analysis). **(B)** Cross-correlation values comparing cortical (CH05) vs. subcortical (CH13) signals from 16 individual recordings across five FL animals indicating the negative shift in cross-correlation near the site of the FL microgyrus. There was a positive correlation between cross-correlation values and distance to lesion (*P* < 0.05, Pearson’s correlation value = 0.525).

Cross-correlation values were computed from nine experimental animals (4 control/5 FL). Micro-electrode position for these nine animals, in comparison to the location of the nearest microgyrus in FL animals, is demonstrated from the cortical reconstruction diagrams in Figure 1F. Multifactorial ANOVA indicated significant main effects across both electrode and treatment group variables (*P* < 0.05) with a significant interaction between the two groups (*P* < 0.05). Specifically, we focused on the cross-correlation differences between treatment groups. A Holm-Sidak *post hoc* analysis indicated significant differences in cross-correlation values between control and FL animals for all subcortical electrodes (i.e., 13–16), as demonstrated in Figure 11A. The lowest correlation values were observed in electrodes 13 and 14 of FL animals with average values in both electrodes near 0. These values represent recordings from fiber tracks of the external capsule (see Figure 3B). A significant reduction in average correlation values was also observed in the deeper subcortical recordings as well (i.e., channel 15, 16). Further analysis also indicated a significant relationship between cross-correlation value and distance to the lesion (Figure 11B, *P* < 0.05, Pearson’s correlation coefficient = 0.525). Specifically, strong negative cross-correlation values (−0.43 to −0.87) were observed within 1200 microns of the lesion. Taken together, these data suggest a disruption of synchronous LFP activity between cortical and subcortical brain structures occurs near the site of the FL microgyrus. The resulting loss of extracellular signal synchrony thus served as a physiological biomarker to indicate that the recording electrode was approaching the site of the cortical malformation.

### FL-Induced SWDs

**Figure 12:**
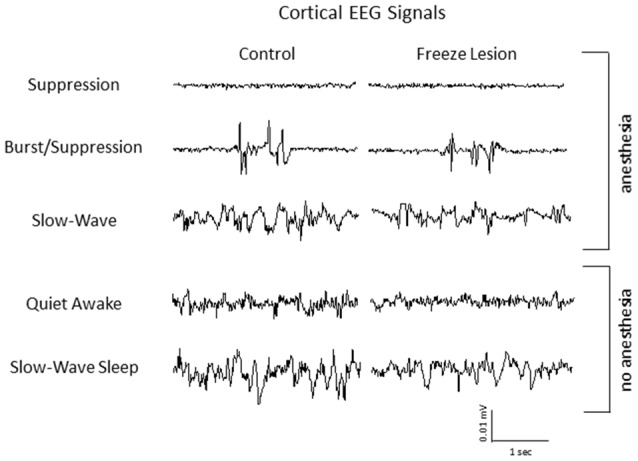
**Representative intracranial electroencephalogram (EEG) recordings from anesthetized and awake animals.** In anesthetized animals, EEG exhibited a similar transition from suppression, burst/suppression, to slow-wave activity as observed from the extracellular recordings (i.e., Figure 2). Anesthetized EEG signals are also compared to non-anesthetized EEG during both quiet awake and slow-wave sleep behavioral states.

**Figure 13:**
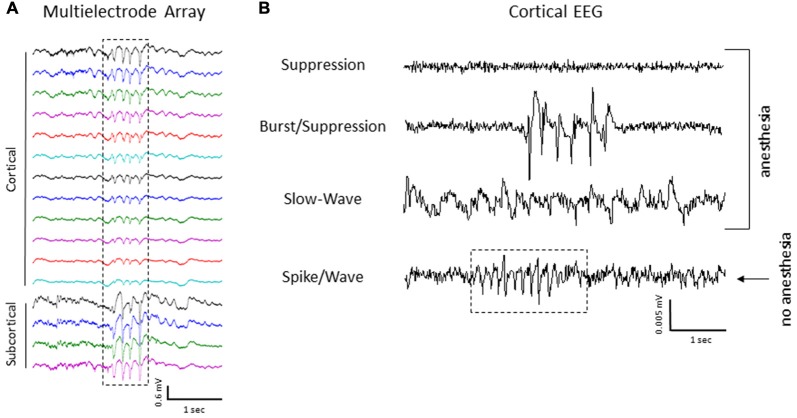
**Spike/wave burst activity and intracranial EEG. (A)** Example of a spike/wave pattern observed during a burst/suppression event from extracellular micro-electrode recordings in a FL animal (indicated by box). **(B)** Cortical EEG recordings from the S1 region of the same animal shown in panel **(A)**. The cortical EEG recording exhibited a similar waveform pattern across the three anesthetic states, including spike/wave patterns during burst activity as compared to extracellular recordings. A 24 h continuous recording in the unanesthetized animal also indicated the presence of repetitive spike-wave discharges (SWDs), occurring predominately during slow-wave sleep (indicated by box in lower trace).

Intracranial EEG recordings were obtained in mice (3 control/4 FL) under both anesthetized and non-anesthetized states for comparison to extracellular linear electrode array recordings in head-fixed anesthetized mice. Representative EEG recordings from the ipsilateral S1 cortex of control and FL animals are shown in Figure 12. EEG signals from all animals progressed from a state of signal suppression, burst/suppression, to slow-wave activity as anesthesia dose was gradually lowered (3.0%–0.5%; Figure 12), similar to that observed in extracellular recordings (i.e., Figure 2). Burst activity presented as brief, abrupt, arrhythmic increases in signal amplitude, observed at isoflurane levels ranging from 0.5% to 2.0% in both control and FL animals. Anesthetized EEG recordings were also compared to non-anesthetized EEG from the same animal during quiet awake and slow-wave sleep activity (Figure 12). In addition, FL animals exhibited SWDs (Figure 13) that were absent in control animals. The non-convulsant SWD pattern that occurs in unanesthetized animals exposed to neonatal FLs to the S1 cortex has recently been characterized in this model (Sun et al., 2016) and occurred in all 4 FL animals in the current study. In one animal with seizures, both extracellular and EEG recordings were obtained as demonstrated in Figure 13. In extracellular recordings, brief instances of spike/waves, lasting less than 1 s in duration, occurred during periods of burst activity, typically bounded by periods of arrhythmic activity (Figure 13A). An average of 4–7 spikes/s were observed per event and the spike/wave pattern occurred simultaneously across all electrodes, with the highest amplitude in the superficial cortical layers and in subcortical regions. Spike/waves were not observed during suppression or slow-wave anesthetic states from the extracellular recordings. Cortical EEG recordings from the same animal are shown in Figure 13B for comparison. A rhythmic pattern of spikes or spike/wave activity was also observed throughout the cortical EEG during burst activity (3–5 spikes/s) but was not observed during suppression or slow-wave anesthetic states. In the unanesthetized state, a typical SWD is shown in the bottom row from this same animal (Figure 13B). All SWDs in the unanesthetized state occurred during slow-wave sleep had an average spike rate of 7–9 spikes/s, about twice that observed during burst activity in the anesthetized state. Overall, spike/waves were observed in the extracellular recordings of 3/9 FL animals though presented as the dominant waveform in only one animal (i.e., Figure 13A). In the other two FL animals, the predominant burst pattern consisted of arrhythmic signal fluctuation with occasional spikes or spike/wave bursts. A higher incidence of spike/waves may have been observed with longer recording sessions. Total recording time of extracellular signals was typically less than 10 min/animal as compared to the 24 h continuous EEG recordings in FL animals that have been reported to consist of a high prevalence of SWDs (Sun et al., 2016).

## Discussion

Similar to previous reports (Sun et al., 2016), EEG recordings from unanesthetized animals indicated that neonatal FLs to the S1 cortex induced robust SWDs. The FL-induced spontaneous 7–9 Hz SWD pattern was similar to the spontaneous SWDs observed in inbred rodent strains including genetic models of absence epilepsy (Danober et al., 1998; Coenen and Van Luijtelaar, 2003; Letts et al., 2014) with an ictal presentation similar to the continuous spike-wave pattern that occurs during slow-wave sleep in the human epileptic condition described as an electrical status epilepticus during slow-wave sleep (Sun et al., 2016). Given the robust seizure profile associated with this model, the main goal of the current study was to characterize the spatiotemporal structure of the spontaneous paroxysmal epileptiform activity using high resolution extracellular recordings obtained with linear microelectrode arrays with a particular aim to characterize possible *in vivo* hyper-excitabilities in the anesthetized brain that may serve as biomarkers for defining the epileptogenic zone.

The progression of the brain from wakefulness to a deep coma-like state during anesthesia has been well characterized from electrophysiological recordings in animal models (Silva and Antunes, 2012) and the clinical population (Purdon et al., 2015). Initially, during light anesthesia, the brain enters a state of slow-wave activity similar to slow-wave sleep. A loss of consciousness soon follows concomitant with a reduction in physiological responses and eventual suppression of the EEG waveform as the anesthetic dose is increased. Though general anesthetics differ in their specific mechanistic brain targets they are believed to act through a general suppression of neuronal activity and consequent shift in the excitatory/inhibitory balance of the brain (Amzica, 2009). An interesting consequence is the induction of burst/suppression activity during the transition from slow-wave to full EEG suppression that has been associated with a stronger decrease in inhibition as compared to excitation (Ferron et al., 2009). In fact, the interim phase of general anesthesia associated with burst/suppression has been described as a “hyperexcitable” brain state directly linked to reduced cortical inhibition, possibly involving the suppression of cortical interneuron activity (Kroeger and Amzica, 2007; Amzica, 2009; Ferron et al., 2009).

In the current study, three anesthetic states were defined based on the dominant extracellular waveform observed: suppression, burst/suppression, or slow-wave activity. All three waveforms were observed in control and FL animals in concordance with the depth of isoflurane anesthesia. Spike/wave patterns were also observed in the extracellular waveforms but were dependent on the anesthetic state of the animal. No ictal activity was observed during the deepest anesthesia levels evaluated. This was not unexpected as this level of anesthesia is associated with a strong suppression of extracellular waveforms as well as neuronal firing (current data; Friedberg et al., 1999; Lukatch et al., 2005). In fact, isoflurane is used in emergency cases of refractory status epilepticus to suppress seizures when other treatment options have failed (Kofke et al., 1989; Trinka et al., 2015). We did expect to observe ictal events at lighter levels of anesthesia, in particular during induction of slow-wave activity, as the SWDs observed from cortical EEG recordings in this model occur predominately during slow-wave sleep (Sun et al., 2016). However, we failed to observe any ictal events at the lightest anesthesia level evaluated (0.5% isoflurane) though this anesthetic state was associated with the induction of a prominent slow-wave pattern in the extracellular waveform. The lack of any paroxysmal activity during the suppression or slow-wave anesthetic states may be related to the limited recording sessions. Total recording time of extracellular experiments consisted of minutes as compared to the 24 h EEG recordings performed in unanesthetized animals. However, another likely scenario may be related to the shift in excitatory to inhibitory tone of the anesthetized brain. During isoflurane levels ranging from 0.5% to 2.0% a significant increase in burst/suppression was observed in FL animals. In fact, the main difference between the treatment groups was an increase in incidence and severity (total power) of burst/suppression activity in FL animals. As such, the main focus of this discussion will be on burst/suppression and the mechanistic implications that can be inferred from the increased prevalence of burst activity in the FL brain.

Burst/suppression is an electrographic pattern of signal suppression interrupted by brief periods of high-voltage slow or sharp waves typically associated with a diverse spectrum of pathological disorders including coma, brain trauma, drug intoxication, hypothermia, and cortical tumors (Amzica, 2009, 2015) but can also be induced pharmacologically, particularly with anesthetics such as isoflurane, propofol, and barbiturates (Friedberg et al., 1999; Lukatch et al., 2005; San-juan et al., 2010; Sellers et al., 2013). In all cases, burst/suppression is associated with loss of consciousness (Amzica, 2015) though the specific etiology can be associated with distinct waveform patterns (Kenny et al., 2014). The brief periods of burst activity that occur during burst/suppression are associated with depolarizing intracellular potentials of individual neurons at the microscopic scale (Steriade et al., 1994) and with an overall hyperexcitable cortical network at the macroscopic scale (Kroeger and Amzica, 2007). Given that the adult FL brain is also associated with an increased hyperexcitability (Jacobs et al., 1996, 1999a; Luhmann and Raabe, 1996; Roper et al., 1997; Redecker et al., 1998), it is not surprising that FL animals exhibited an increase in burst/suppression under isoflurane anesthesia. Essentially, microgyric lesions within the S1 cortex appear to predispose the brain to hyperexcitable bouts of burst/suppression (i.e., higher burst incidence) as well as increase the severity of each event (i.e., increased total spectral power) not unlike the susceptibility of the epileptic brain to pro-convulsant stimulants. However, not all FL models are sensitive to pro-convulsants. A reduced seizure threshold was not observed in the pentlyenetrazol model (Kellinghaus et al., 2007) or after amygdala-induced kindling (Holmes et al., 1999) following neonatal FLs in rats. However, a reduced threshold for hyperthermia induced seizures was reported following neonatal FLs (Scantlebury et al., 2004) and in amygdala kindled seizures following prenatal FLs (Takase et al., 2008) in rats. In each report, unilateral FLs were induced in the frontoparietal cortex though the morphological characteristics of the dysplastic cortex and stereotaxic location of the microgyrus varied across studies. In addition, the EEG seizures associated with S1 FLs in the current model differ from the polyspike seizures observed following bilateral cortical FLs induced in rats at day E18 (Kamada et al., 2013) or the lack of seizures reported in other FL models (Kellinghaus et al., 2007). Taken together, these reports indicate that the location of the microgyrus and extent of the FL-induced injury may have an important impact on the induced epileptogenicity of the brain. The strong thalamocortical connectivity of the barrel system may be particularly sensitive to FL-induced generation of SWDs. A comprehensive comparison of the epileptic outcome in reference to differences in the location or severity of experimental FLs has not been reported to date though it could have clinical significance for determining optimal regions of surgical resection in cases of intractable epilepsy.

The initiation of spontaneous burst/suppression activity is purported to involve subliminal stimuli, of internal or external origin, or a consequence of ongoing subcortical activities, though each burst event is limited in duration and is followed by a refractory period (Kroeger and Amzica, 2007). In the current study, control and FL animals exhibited a decrease in the rate of burst/suppression as anesthesia level was increased, though there was no significant difference in burst rate between control and FL animals. This lack of change in the periodicity of burst/suppression activity may indicate a stable metabolic state of the FL brain as compared to control animals. This is supported by studies indicating that the hyperexcitability of experimental FCD does not alter glucose metabolism (Redecker et al., 1998). Kroeger and Amzica (2007) reported that burst/suppression is coupled to a depletion of extracellular Ca^++^ levels limiting synaptic activity and suggested that the refractory period following an individual burst is related to the ability of ATP pumps to restore Ca^++^ levels. Interestingly, global changes in extracellular Ca^++^ levels were correlated to the level of isoflurane (Kroeger and Amzica, 2007), that may explain the change in burst rate observed across anesthetic dose. In addition to the general suppression of neuronal activity associated with anesthetics, a more complex mode of action is linked to the large-scale modulation of neural networks across brain regions (Cimenser et al., 2011; Lewis et al., 2012; McCarthy et al., 2012). Although the fundamental circuitry of the mammalian cortex is remarkably similar across brain regions, significant differences in the specific details of individual computational units exist that may account for differential effects of anesthesia across brain region and cortical layer (Tan, 2015; Miller, 2016). In the current study, laminar recordings of extracellular signals indicated significant changes in spectral power values across anesthetic state but not between cortical lamina of the S1 cortex though there was a trend for increased power levels in the superficial layers as observed in some animals. In comparison, Sellers et al. (2013) described layer IV of the primary visual cortex in ferrets to be largely resistant to changes in anesthesia level as compared to the pronounced modulation of spectral power in the supragranular and infragranular layers while the primary frontal cortex exhibited dramatic increases in spectral power across both anesthesia level and cortical lamina. Taken together, these studies indicate that anesthetics such as isoflurane do not induce homogeneous changes in cortical signals across brain regions and appear to disrupt information processing in a region-specific manner.

Several forms of neurological disease, including brain injury and epilepsy have been associated with disruption of normal brain electro-oscillatory behavior (Pevzner et al., 2016). In the current study, the spectral profile of burst activity was similar to slow-wave activity in both control and FL animals with the majority of spectral power reflected in the lower frequencies (1–20 Hz) and highest values in the delta (1–4 Hz) and theta (4–8 Hz) bands. Slow-wave oscillations are a common feature of non-REM (i.e., slow-wave) sleep, anesthesia, and are commonly observed in cortical slices (Tan, 2015). In fact, slow waves may represent global changes in information flow that entrain much of the cortex (Massimini et al., 2004) or may represent distinct features that shape activity in specific functional circuits (Kenet et al., 2003). Synchronization of specific brain oscillations are a key factor driving communication between brain regions (Fries, 2015) though there is considerable variation in slow-wave synchrony within the cortex (Mohajerani et al., 2010) that is further disrupted during anesthesia (Imas et al., 2006; Cimenser et al., 2011). In the current study, burst activity near the S1 cortex of control animals occurred uniformly across cortical lamina and exhibited high cross-correlation values with minimal phase shift across the vertical extent of the linear microelectrode, encompassing both cortical and subcortical regions. In contrast, signal synchrony of cortical vs. subcortical regions near the microgyrus was significantly lower with strongly negative cross-correlation values within 1200 microns of the microgyrus. This disruption in signal synchrony in dysplastic cortical vs. the underlying subcortical regions may be directly related to altered thalamocortical projections thought to be responsible for the hyperexcitable innervation of regions near the microgyrus (Jacobs and Prince, 2005) although the direct impact that disrupted signal synchrony between cortical and subcortical regions would have on the possible pathogenicity of SWDs in not clear. Current theories support a “cortical focus” for the initiation and propagation of the spontaneous SWDs observed in animal models of absence epilepsy though the thalamus plays an important part in the amplification and maintenance of rhythmic SWDs (Meeren et al., 2005). In fact, intact thalamic sub-circuits are critical components for the generation of SWDs, as demonstrated from the suppression of SWDs following thalamic lesions in a WAG/Rij rats (Meeren et al., 2009). As indicated in the current study, microgyric lesions in the somatosensory cortex could have a direct impact on the subversion of normal thalamocortical rhythms towards promotion of SWD patterns though a cortical focus within the hyperexcitable tissue surrounding the microgyrus is also likely. Future research will be necessary to link the critical features related to the pathophysiology of the dysplastic S1 cortex to generation of SWDs.

In summary, the present findings indicated significant electrophysiological changes in mouse brain exposed to neonatal FLs in the S1 somatosensory cortex that were dependent on anesthetic state. Three main findings of the current study include: (1) FL animals had a higher incidence of burst/suppression activity with significantly higher spectral power values than controls; (2) LFPs were strongly out of phase between cortical and subcortical regions near the site of the FL induced microgyrus; and (3) “hyperexcitable” burst activity in FL animals shared similar spike/wave components with the SWDs observed from cortical EEG recordings in unanesthetized animals. The hyperexcitability associated with anesthesia-induced burst/suppression may serve as a useful medium for studying aberrant network activity in the seizurogenic brain that may extend to other brain injury models as well. Furthermore, the utilization of microelectrode arrays allows the recording of broad spatiotemporal changes in electrophysiological signals of the dysplastic cortex and underlying subcortical regions. Assuming the observed hyperexcitability is confined to or initiates from the epileptogenic zone of the FL brain, the distinct spectral characteristics of burst activity, as indicated in the current study by altered synchrony of field potentials across cortical/subcortical boundaries, may serve as viable biomarkers for defining regions of epileptogenesis with direct clinical value. Additional studies will be needed to determine if burst activity, or in fact SWDs, initiate from the dysplastic S1 cortex in the current model and to what extent seizurogenic activity may originate from other cortical regions exposed to FL injury. With the commercial availability of multi-dimensional arrays, future studies will greatly expand our knowledge of the disrupted neural networks that drive aberrant brain neurophysiology with the ability to map entire networks of pathological brain tissue in the epileptic brain.

## Ethics Statement

The protocol was approved by the Institutional Animal Care and Use Committee of the University of Wyoming.

## Author Contributions

AJW and Q-QS contributed to the design, writing, and editing of the manuscript. AJW performed the multi-electrode array recordings and data analysis. CZ performed the EEG recordings and related data analysis.

## Funding

Funding for this research provided from NINDS/NIH grant 5R01NS094550. CZ is supported by a research studentship from 5P20GM103432 (NIGMS).

## Conflict of Interest Statement

The authors declare that the research was conducted in the absence of any commercial or financial relationships that could be construed as a potential conflict of interest.
